# A Pilot Program to Teach Pharmacy Students Practical Skills to Navigate Drug Insurance Benefits

**DOI:** 10.3390/pharmacy10010023

**Published:** 2022-01-27

**Authors:** Camlyn Masuda, Tony Huynh, Veronica Wong, Colette DeJong, Chien-Wen Tseng

**Affiliations:** 1 Department of Pharmacy Practice, Daniel K. Inouye College of Pharmacy, University of Hawaii at Hilo, Hilo, HI 96720, USA; tonyhuyn2016@gmail.com (T.H.); wongvjy@gmail.com (V.W.); 2 Department of Medicine, University of California, San Francisco, CA 94143, USA; colette.dejong@ucsf.edu; 3 Department of Family Medicine and Community Health, John A. Burns School of Medicine, University of Hawaii, Honolulu, HI 96813, USA; cwtseng@hawaii.edu

**Keywords:** pharmacy education 1, formulary 2, out-of-pocket cost 3, insurance 4, pharmacy practice 5

## Abstract

Pharmacists must be able to navigate prescription drug coverages to help providers and patients reduce out-of-pocket costs. Traditionally, curricula on drug insurance benefits rely on lectures and lack a practicum that offers students hands-on experience with determining formulary and cost-sharing information. An activity for pharmacy students to update a free public website that summarizes formularies and copayment requirements across major insurers was piloted. Pharmacy students were trained to locate online formularies and identify a drug’s coverage tier, step therapy, prior authorization, and cost-sharing during a 6-week experiential rotation. Students checked formularies from six insurance plans for 250-plus drugs across 15 health conditions. Graduates were surveyed (74% response rate) about the activities’ impact on their learning and ability to navigate drug benefits. Respondents rated the training as helpful in learning whether a drug was covered (100%), or required step therapy or prior authorization (100%). The majority of graduates reported being able to look up formulary coverage (90%), step therapy or prior authorization (90%), and copayment requirements (65%). Our innovative skills-based pilot activity was effective in teaching pharmacy students to navigate insurance formularies, which is essential for helping patients access medications.

## 1. Introduction

In the United States, out-of-pocket costs and lack of insurance coverage are among the top five reasons for non-adherence to medications [1,2]. In 2019, nearly 3 in 10 Americans reported not using medications as prescribed because of out-of-pocket costs [3]. Pharmacists who are trained to navigate drug benefits, formularies, and coverage requirements are uniquely positioned to help lower patients’ cost-sharing and increase treatment adherence [4]. Common health conditions—like asthma and diabetes—often have several therapeutic options within each treatment class, and the cost and coverage of specific drugs can vary widely between insurance plans depending on contracts negotiated with competing pharmaceutical companies [5,6]. As a result, providers can find it challenging to know which drug is covered for each patient and may inadvertently prescribe non-formulary or non-preferred drugs with higher copayments when less expensive, effective options exist [7]. With training and experience, pharmacy students who enter a career in retail or clinical settings can help prescribers and patients determine which evidence-based medications are covered and/or have lower cost-sharing [8,9,10,11,12].

The importance of pharmacy training on insurance, drug costs, and access to care is recognized by the Accreditation Counsel for Pharmacy Education (ACPE) which requires pharmacy schools in the United States curricula to include healthcare systems, reimbursement models, and patient advocacy [13]. A task force that included the National Community Pharmacists Association (NCPA) created a list of entry level pharmacist’s competency requirements based on surveys of community pharmacy employers and included knowledge of formularies, prior authorization, and step therapy [14]. Teaching pharmacy students how to help patients navigate drug insurances is also part of patient-centered care, an educational outcome recommended by the Center for the Advancement of Pharmacy Education (CAPE) [15]. CAPE outcomes are targets which pharmacy curricula in the should meet to develop practice-ready pharmacists [15]. In our literature review of pharmacy curricula to train students about insurance and drug formularies, most courses rely on didactic lectures about healthcare policy or management or activities on formulary management. Such curricula generally lack a practicum component to teach students hands-on, practical skills needed to navigate drug insurance plans and formularies [16]. There are some curricula that train students to help patients enroll in drug insurance plans, such as Medicare (United States government-funded health and drug insurance programs for those who are 65 years and older, have disabilities or with End-Stage Renal Disease), and provides some information about tiering system and Medicare program structure [17,18]. There are no examples in the literature of an experience-based activity that trains pharmacy students to navigate multiple drug benefits and determine formulary coverage, or cost-sharing for patients and measured their ability to complete these activities after graduation.

In this study, we piloted an elective hands-on training activity for pharmacy students to learn these skills. Students were first trained to review formularies of major insurance plans and collaborate in updating a free statewide clinician-facing website that summarized drug coverage for common health conditions, then the students were then asked to update the formularies for 1–3 insurance companies offered in Hawaii during a 6-week period. Graduates of the pilot program were surveyed as to (1) whether the training taught them useful pharmacy skills, and (2) their self-reported ability to determine formulary coverage and cost-sharing for their patients.

## 2. Materials and Methods

The pilot included two to three pharmacy students from the University of Hawaii at Hilo Daniel K. Inouye College of Pharmacy, every two to three months to engage in a six-week activity to update the www.PrescribingGuide.com, a free, non-profit, grant-funded, statewide prescribing resource for clinicians in Hawaii. Only fourth-year pharmacy students who were in an Advanced Practice Pharmacy Experience (APPE) ambulatory care course (usually 2–3 students per 6-week course) with Dr. Masuda as the preceptor from 2016 to 2019 completed the updates of the formulary as part of their course if recent formulary updates/changes were made by the insurance. All students who participated in updating the website were eligible to complete the voluntary survey after graduation.

The pharmacy students receive 2–4 h of lectures on insurance plans and formulary terminology in the first year of the program at the University of Hawaii at Hilo Daniel K. Inouye College of Pharmacy in the Introductory to Pharmacy Practice course. In the third year of the curriculum, the students learn about formulary management and the process for selecting medications to be on a formulary in the Healthcare systems class. To assist the students with applying and advancing the skills learned in their first and third year of pharmacy school, a training activity was designed to target three practical skills: (1) navigating drug insurance types (commercial, Medicaid (state-funded health and drug insurance for those with limited income), (2) understanding how drug benefits are structured (deductibles, copayments), and (3) understanding drug formulary criteria (tiers, step therapy, prior authorization, preferred versus non-preferred coverage). The training met ACPE requirements and CAPE educational outcomes on medication use systems management (managing patient healthcare needs using human, financial, technological, and physical resources) and patient advocacy (helping patients access financial, technological, and physical resources to optimize the safety and efficacy of medication use systems) [13,15].

**Figure 1:**
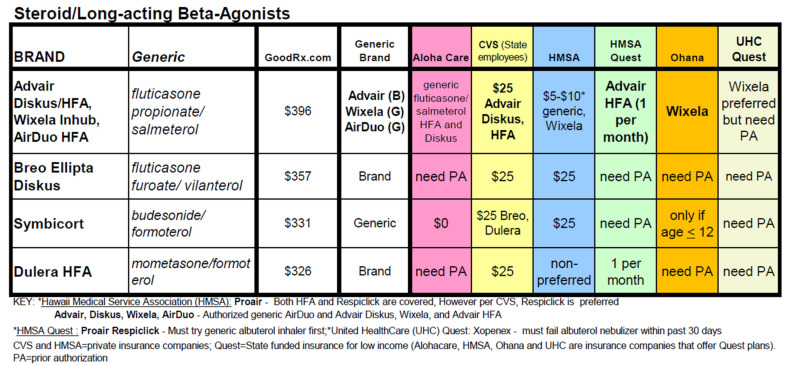
Example of a table of formulary status of drugs on the www.prescribingguide.com website.

The University of Hawaii at Manoa John A. Burns School of Medicine, Department of Family Medicine and Community Health sponsors the website with the goal of improving medication access to patients. The Prescribing Guide summarizes formulary and drug benefit information from six major local health insurers for over 250 drugs treating 15 common health conditions, including asthma, diabetes, hypertension, hypercholesterolemia, and depression (Figure 1). Prescribers can identify at a glance whether a drug is covered by a particular plan, is preferred or non-preferred, belongs in a lower versus higher copayment tier, or requires step therapy or prior authorization. Drugs are grouped by health condition and treatment class, so that prescribers can quickly identify a covered alternative if a drug is non-formulary or non-preferred. The Prescribing Guide is updated six times per year or more frequently if there are formulary changes that have high clinical relevance.

**Figure 2:**
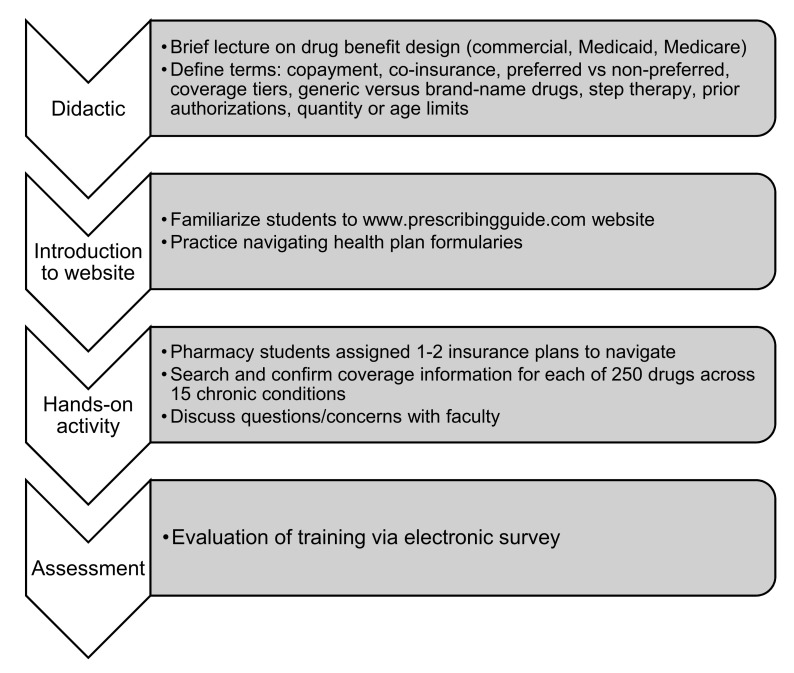
Pharmacy student training workflow.

Figure 2 highlights the pharmacy student’s workflow for the pilot program. Course participants first attended an introductory didactic lecture (15–20 min long) on drug benefit design for commercial, Medicaid, and Medicare plans. Next, for the experiential learning portion of the pilot program, students were trained to find drug insurance websites (links to the insurance websites are also available on the www.PrescribingGuide.com website) and navigate plans’ online formularies. Students researched each of the 250-plus drugs included in the Prescribing Guide, to determine the formulary coverage, tier level, whether a drug was preferred or non-preferred, and if step therapy or prior authorization was required. If information on coverage was not available online, such as for glucometers or spacers for inhalers, students were trained to call health insurance plans directly to inquire about coverage. For drugs with step therapy, prior authorization quantity, or age requirements, students documented the criteria for meeting those conditions—such as which drugs needed to be tried first or the number of pills covered per month. Coverage changes detected by students were reviewed and checked by faculty to confirm accuracy before being updated on the www.PrescribingGuide.com website. Investigators (Masuda, Tseng, and Huynh) were available at all times to coach and supervise students in this activity.

To evaluate the program, the 42 graduates who completed the pilot training between 2016 and 2019 were emailed an electronic survey between March to August 2020. Participants’ email information was collected to ensure responses were unique. The survey addressed three areas: (1) participants’ opinion on the importance of learning about drug insurance plans and cost-sharing for patients, (2) whether the training activity was helpful in improving their knowledge of formularies, prior authorizations, step therapies, and cost-sharing for patients, and (3) their self-reported ability to look up formulary coverage information and copayments for their patients. Responses were measured using a 5-point Likert scale. Graduates also reported their current field of practice. This study was approved by the University of Hawaii Office of Research Studies (#2019-01059).

## 3. Results

Thirty-one of 42 pharmacy graduates who participated in the pilot completed the evaluation survey (response rate 74%). The majority of respondents worked in retail (39%), inpatient (23%), specialty pharmacies (10%) or ambulatory care (6%) settings. Table 1 includes gender information and the areas of pharmacy practice of the responders.

Table 2 presents all the questions on the survey and response rates. In evaluating the program, all respondents considered it important for pharmacy students to learn about drug insurance (55% very important, 45% important). All respondents rated the hands-on experience of working on www.PrescribingGuide.com as helpful in skill-building, such as determining whether a drug was covered by a formulary (70% very helpful, 30% helpful) and whether a drug required step therapy or prior authorization (67% very helpful, 33% helpful). In terms of practical skills, at least 90% of respondents reported that they knew how to look up whether a drug was covered by a patient’s formulary (23% strongly agree, 68% agree) and required step therapy or prior authorization (16% strongly agree, 74% agree).

With respect to patient cost-sharing, 94% of respondents considered it important to learn about patients’ out-of-pocket cost for prescriptions (39% very important, 55% important) (Table 2). A total of 85% of respondents felt the training activity helped them learn what a patient’s copayment would likely be (33% very helpful, 52% helpful, 7% neutral, 7% not helpful) and 65% self-reported that they knew how to look up copayment information (13% strongly agree, 52% agree, 23% neutral, 13% disagree).

## 4. Discussion

Our pilot study highlights the effectiveness of a skills-based training activity to instruct pharmacy students on drug benefits and out-of-pocket costs. In a post-course evaluation, diverse participants from retail, inpatient, and ambulatory pharmacy backgrounds reported that the training on formularies and out-of-pocket costs was an important part of their pharmacy education. They rated the hands-on experience as very helpful in teaching practical skills, and the majority of graduates reported that they would be able to look up formulary coverage, step therapies and prior authorization requirements. A majority of the respondents were working in retail settings and using the newly developed skills would be the most helpful in helping patients find alternative formulary medications. These findings suggest the importance of hands-on training activity to teach pharmacy students to navigate drug benefits for their patients.

Our pilot was specific to updating www.PrescribingGuide.com for residents of Hawaii, and was extensive in that students reviewed formulary coverage for over 250 drugs treating over 15 health conditions across 6 insurers. However, this training activity can be adapted to a shorter practicum in the United States and other countries that do not have socialized medicine by targeting a concise group of drugs with highly variable formulary coverage (e.g., asthma inhalers or diabetes therapies), and by reviewing just two or three insurance plans with formularies available online. The activity can also be adapted to countries with socialized medicine by focusing on the government’s drug formulary, allowing students to search for status of drugs in a class to find a drug that is covered and discuss reasons for why the drug is covered. In our experience, it usually took students several iterations with supportive coaching and feedback to be consistently accurate in determining drug coverage information. It was also important that students had the chance to practice these skills for drugs from multiple health conditions and across different insurance types that differ in their coverage models (commercial, Medicaid). Our main objective was that pharmacy students learn the practical skills that they will need in their pharmacy careers. Our findings can help other pharmacy education leaders design their own hands-on curriculum to teach these skills.

Pharmacists are often on the frontline when patients pick up their prescriptions and are surprised by high copayments. Although most respondents felt that the practicum taught them how to anticipate patients’ cost-sharing requirements, one-third were either neutral or disagreed that they could determine what a patient’s copayment would likely be. We believe this is because insurance plans in the United States often provide information about formulary coverage online but often do not include specific dollar copayments. Exact cost-sharing can vary according to a patient’s plan, other medications, and phase of coverage. Often, accurate information on patient copayment requires a pharmacist to fill the prescription and have the claim adjudicated by insurance [5]. During the rotation, pharmacy students frequently expressed frustration with their inability to determine exact dollar copayments online. In this manner, their experience mirrored the real-world difficulties that pharmacists encounter commonly in practice in the United States. To mitigate these challenges, students were trained to make the most of available cost information to know whether a drug might have lower or higher copayments—for example, identifying whether a drug was generic or brand, and determining its coverage tier and preferred status. To improve medication adherence, coverage and cost information need to be made more easily available to patients, providers, and pharmacists [7].

Limitations of this pilot includes a response rate of 74% and the small sample size of 42. The pilot study did not include a pre-intervention assessment or control group, and relied on respondents’ self-reported assessment of their ability to navigate drug benefits and copayments. In future iterations of the pilot, we hope to design objective assessment tools to measure students’ fluency and accuracy in researching drug coverage, step therapy and prior authorization requirements, and copayments. We are also exploring ways to teach students about patients’ cost-sharing for prescriptions and to expand the practicum to all students during their APPE rotations. Another consideration is to incorporate the activity earlier in the curriculum, such as the first year of pharmacy school, which would provide a baseline knowledge an application to better prepare students for the progression to making decisions on formulary status for medications.

## 5. Conclusions

An experienced-based pilot curriculum on drug insurance gave pharmacy students the practical skills they need to navigate drug coverage and cost information. Such training allows future clinical pharmacists to identify covered medications, flag medications requiring prior authorization or step therapy, and pinpoint alternative therapies. Incorporating these experiences into pharmacy school is essential to producing pharmacists who can help patients and providers identify effective as well as affordable drugs and ensure that graduating pharmacy students have the entry-level skills that employers are expecting.

## Figures and Tables

**Table 1:** Demographics of pilot participants.

**Sex**	
Male	17 (55%)
Female	14 (45%)
**Current pharmacy practice**	
Retail	12 (39%)
Inpatient	7 (23%)
Ambulatory care/Medication Therapy Management	2 (6%)
Specialty	3 (10%)
Nuclear pharmacy	1 (3%)
Other	6 (19%)

**Table 2:** Pharmacy graduates’ responses on the importance and usefulness of learning skills to navigate drug insurances (n = 31) *.

**1. Is It Important to Learn about …**			
	Drug insurance, formularies, and how to find out if a drug is covered or not?		Patients’ out-of-pocket cost for prescriptions (copayments, retail prices)
Very important	55%		39%
Important	45%		55%
Neutral	0%		6%
Unimportant	0%		0%
Not at all important	0%		0%
2. Did the Training Help You to Learn … **			
	Whether a drug is covered on a plan’s formulary?	Whether a drug requires step therapy or prior authorization?	What a patient’s copayment will likely be?
Very helpful	70%	67%	33%
Helpful	30%	33%	52%
Neutral	0%	0%	7%
Not helpful	0%	0%	7%
Not at all helpful	0%	0%	0%
**3. I Know How to Look Up…**			
	Whether a drug is covered on a plan’s formulary	Whether a drug requires step therapy or prior authorization	What a patient’s copayment will likely be
Strongly agree	23%	16%	13%
Agree	68%	74%	52%
Neutral	10%	10%	23%
Disagree	0%	0%	13%
Strongly disagree	0%	0%	0%

Key: * Percentages may not add to 100% due to rounding; ** 4 non-respondents.
